# Investigation of different methods in rapid microbial identification directly from positive blood culture bottles by MALDI-TOF MS

**DOI:** 10.1128/spectrum.00638-24

**Published:** 2024-06-28

**Authors:** Dilan Karadağ, Mahmut Cem Ergon

**Affiliations:** 1 Department of Medical Microbiology, Faculty of Medicine, Dokuz Eylül University, İzmir, Turkey; 2 IMD Labor Oderland, Frankfurt (Oder), Germany; Johns Hopkins University, Baltimore, Maryland, USA

**Keywords:** MALDI-TOF MS, direct identification, blood culture, Sepsityper, SDS, centrifugation method

## Abstract

Many methods are being tried for rapid and accurate identification of sepsis-causing microorganisms. We analyzed the performance of three different preparation methods [MBT Sepsityper IVD Kit (Bruker Daltonics GmbH, Germany), sodium dodecyl sulfate (SDS) lysis, and differential centrifugation with protein extraction (Centrifugation +PE)] and compared in standard and Sepsityper modules of the Bruker Biotyper MALDI-TOF MS for direct identification of bacteria from 240 positive blood culture bottles of BACTEC FX (Becton Dickinson, USA). By using the standard module, correct identification at species level (score ≥2) was done in 46.7% of the samples with SDS lysis, 44.2% with centrifugation +PE, and 25.4% with the Sepsityper kit. These ratios at the genus level (score range 1.70–1.99) were 34.6%, 31.3%, and 32.5%, respectively. With SDS lysis (195), more bacteria were identified correctly than centrifugation +PE (181) and the Sepsityper kit (139). A statistically significant difference was found between SDS and the Sepsityper kit and Centrifugation +PE and the Sepsityper kit (*P* < 0.001, both). By using the Sepsityper module, correct identification at species level (score ≥1.8) was determined in 74.2% of the samples with SDS lysis and centrifugation +PE each and 55% with the Sepsityper kit. These ratios at the genus level (score range 1.60–1.79) were 16.3%, 10%, and 19.2%, respectively. SDS lysis (217) had significantly higher identification rates than centrifugation +PE (202) and the Sepsityper kit (178) (*P* = 0.028 and *P* < 0.001). A statistically significant difference was also observed between centrifugation +PE and the Sepsityper kit (*P* < 0.001). Best performance was obtained with SDS lysis among the methods. Although better performance was achieved by using Sepsityper software module, risk of misidentification should not be ignored.

**IMPORTANCE:**

Sepsis is a life-threatening condition, and rapid and accurate identification of the causative microorganisms from blood cultures is crucial for timely and effective treatment. Although there are many studies on direct identification from blood cultures with MALDI-TOF MS, further standardization is still needed. In our study, we analyzed the performance of three different preparation methods and compared by using two analysis modules of the Bruker Biotyper MALDI-TOF MS for direct identification of bacteria from numerous positive blood culture bottles. The literature reports a limited number of studies that compare different preparation methods for direct blood culture identification, processing a large number of blood samples concurrently and evaluating the same samples as in our study. Moreover, although SDS is used very frequently in medical laboratories, there are few studies on direct identification from blood culture bottles. In our study, the highest correct identification rate was observed with the SDS method.

## INTRODUCTION

Sepsis is one of the leading causes of high mortality and morbidity in both adults and children worldwide. According to data from the World Health Organization (WHO), sepsis is estimated to affect approximately 49 million people and causes 11 million deaths globally each year (1).

Rapid identification of microorganisms in a sepsis patient allows for initiation of the correct antibiotic treatment, which not only shortens the patient’s recovery time but also reduces hospitalization duration and other additional costs (2). Blood culture is the gold standard in sepsis, allowing for rapid and accurate identification of microorganisms, which can lower mortality rates and achieve cure with appropriate antibiotic treatment. Early identification of the microorganism is essential for selecting prophylactic antibiotics based on specific bacterial resistance patterns (3). In clinical practice, broad-spectrum antibiotics are often chosen based on the Gram staining characteristics of the bacteria.

In the microbiology laboratory, after receiving a positive blood culture bottle, the identification of the microorganism and the reporting of antibiotic susceptibility tests can take 12–48 hours, depending on the methods used by the laboratory. Therefore, new methods are being explored in laboratories to enable faster identification. Although matrix-assisted laser desorption/ionization time-of-flight mass spectrometry (MALDI-TOF MS) provides a rapid and accurate identification of microorganisms from colony growth on agar plates following blood culture, the time from the positive result in the blood culture bottle to the definitive identification can still exceed 24 hours (4).

The implementation of methods providing direct identification from positive blood culture bottles can reduce the time for microbial identification to approximately 1 hour, and performing rapid antibiotic susceptibility testing (RAST) directly from positive culture bottles, as recommended by the European Committee on Antimicrobial Susceptibility Testing (EUCAST), can further reduce the time to 16–24 hours (5).

There are commercially available kits (6–8) or in-house methods (9–13) for direct identification of microorganisms from blood culture bottles using MALDI-TOF MS. The high cost of commercial kits has led laboratories to develop in-house methods (6, 14). The steps involved in in-house methods are as follows: initially, cellular components are removed through a low-speed centrifugation step. Subsequently, a lysis step is performed using detergents such as SDS, Triton-X, saponin, and Tween-80. Following that, the bacterial pellet is obtained through a higher-speed centrifugation step. Then, a washing step is carried out using ethanol, distilled water, physiological saline, or HPLC-grade ultrapure water. In the final step, the microbial proteomes are extracted, preferably with treatments like formic acid, ethanol, acetonitrile, or acetic acid (9–13).

The MBT Sepsityper is a qualitative *in vitro* diagnostic device consisting of an MBT-CA (Sepsityper) software extension and a reagent kit (MBT Sepsityper Kit US IVD) for use in conjunction with other clinical and laboratory findings to aid in early diagnosis of bacterial and yeast infections from positively flagged blood cultures using the MALDI Biotyper CA System. There are two different workflows when performing the MBT Sepsityper IVD Kit: The Rapid Sepsityper Workflow using Direct Transfer (DT) and extended Direct Transfer (eDT), and the Standard Sepsityper Workflow using the Extraction (Ext). The Sepsityper analysis module uses confidence intervals in lower score ranges compared to the standard module during identification: ≥1.80 high confidence identification (species level), 1.60–1.79 low confidence (genus level), and <1.60 no organism identification possible (8).

The SDS, a harsh ionic detergent, is a very commonly used and effective surfactant in solubilizing most proteins. It disrupts non-covalent bonds within and between proteins, denaturing them, and resulting in the loss of their native conformation and function. The anionic SDS is frequently used in medical laboratories during cell lysis, electrophoresis, Western Blot, and hybridization because it is an effective surfactant in solubilizing most proteins (15).

The aim of the present study was to analyze the performance of three different preparation methods [MBT Sepsityper IVD Kit, sodium dodecyl sulfate (SDS) lysis, and differential centrifugation with protein extraction (PE)] and compare with standard and Sepsityper modules of the Bruker Biotyper MALDI-TOF MS for direct identification of bacteria from positive blood culture bottles.

## MATERIALS AND METHODS

**Fig 1:**
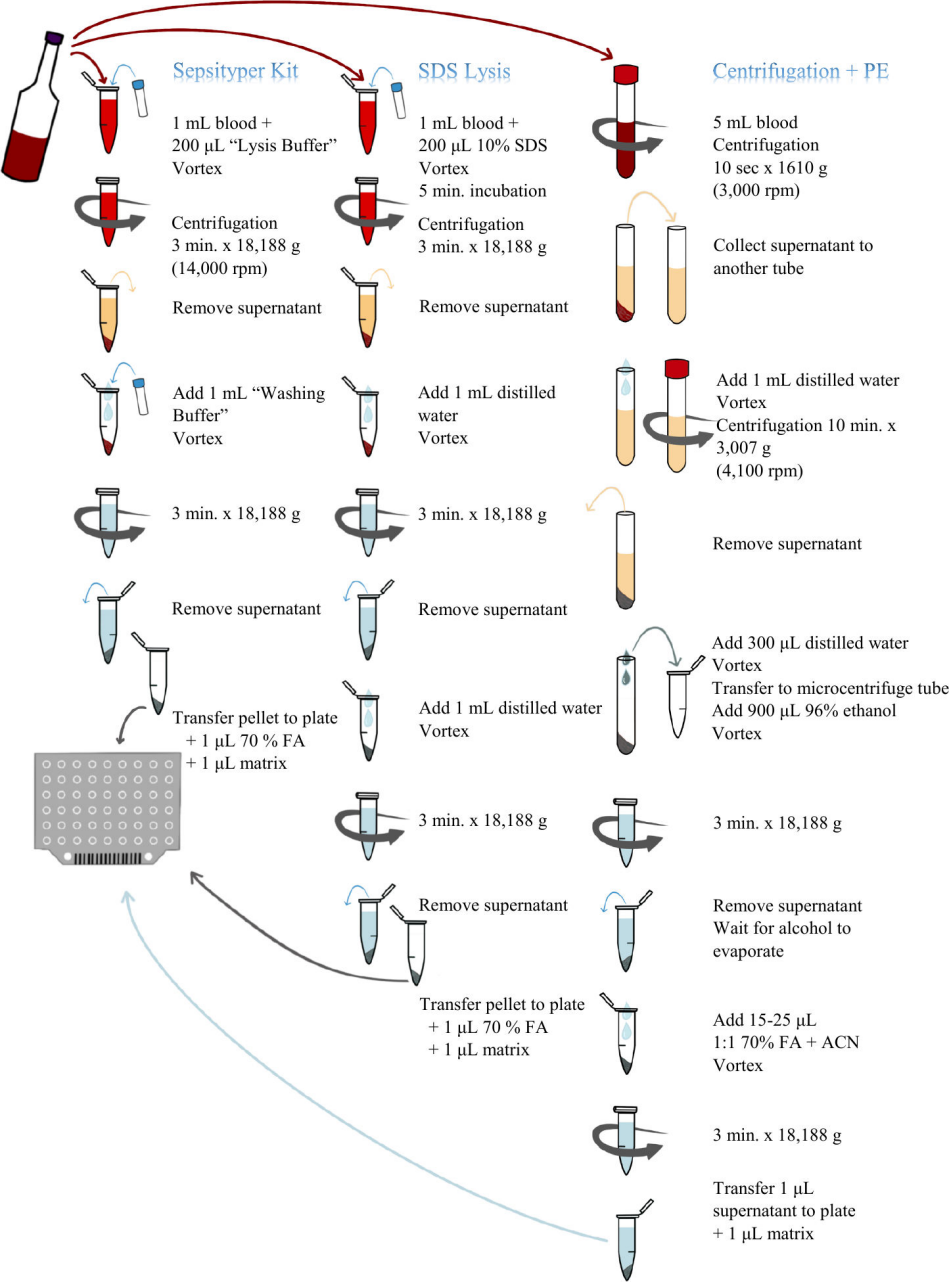
Steps taken to prepare samples for the three methods included in the study. FA, formic acid; ACN, acetonitrile.

In the study, a total of 245 blood culture samples were collected from the Dokuz Eylül University Hospital Central Laboratory Bacteriology Laboratory between December 2022 and March 2023. These samples were incubated in the BACTEC FX (Becton Dickinson, Cockeysville, USA) blood culture instrument. BD BACTEC FX Plus Aerobic, Plus Anaerobic, Lytic/10 Anaerob/F, and Peds Plus/F blood culture bottles were used. The results obtained using the MBT Sepsityper IVD Kit (Bruker Daltonics GmbH, Bremen, Germany), SDS lysis, and differential centrifugation with PE methods were compared with the routine colony identification results by analyzing them on the Bruker Biotyper MBT smart MALDI-TOF MS device’s standard and Sepsityper modules. The three protocols that were used in this study are shown in Fig. 1.

The positive blood culture bottles were subcultured routinely and kept at room temperature. To maintain the efficiency of the study, a maximum of eight samples per day were processed simultaneously with all three methods, provided that the time to growth signal did not exceed 1 day. Repeated growths within the same week and samples showing yeast or polymorphic appearance in Gram staining were excluded from the study.

### Routine colony identification

Following Gram staining analysis, the samples obtained from the positive blood culture bottles were subcultured onto relevant agar plates. Subsequently, the microorganisms that developed on these agar plates after an overnight incubation at 37°C in the respective conditions were identified using the Bruker Biotyper (Microflex LT/SH Smart) MALDI-TOF MS (Bruker Daltonics GmbH, Bremen, Germany). The conventional method was used as a reference method.

### MBT Sepsityper IVD Kit

In order to shorten the identification time and facilitate its application, the “Rapid Sepsityper Protocol” was applied in accordance with the instructions recommended by the manufacturer. One milliliter of blood culture fluid from a positive blood culture bottle was transferred into a microcentrifuge tube. Two hundred microliters of lysis buffer was added, and it was vortexed thoroughly for approximately 10 seconds. The tube was centrifuged at 18,188 g (14,000 rpm) for 3 minutes at room temperature (samples with poorly separated pellets were vortexed and centrifuged again). The supernatant was discarded using a pipette. One thousand microliters of washing buffer was added, and it was vortexed thoroughly. The tube was centrifuged at 18,188 g for 3 minutes. The supernatant was discarded. The pellet was transferred to a MALDI target plate (Bruker Daltonics GmbH, Bremen, Germany) by using a wooden stick (samples that could not be picked up with a stick were transferred with a 1-µL pipette). It was covered with 1 µL of 70% formic acid and allowed to dry at room temperature.

### SDS lysis

One milliliter of blood culture fluid from a positive blood culture bottle was transferred to a microcentrifuge tube. Two hundred microliters of a 10% SDS solution was added, vortexed thoroughly for approximately 10 seconds, and incubated for 5 minutes at room temperature. After incubation, the tube was centrifuged at 18,188 g (14,000 rpm) for 3 minutes at room temperature (samples with poorly separated pellets were vortexed and centrifuged again). The supernatant was discarded by using a pipette, and the pellet was washed with 1 mL of distilled water and vortexed. The tube was centrifuged at 18,188 g for 3 minutes. The washing and centrifugation processes were repeated once. The supernatant was discarded by using a pipette. The pellet was transferred to the target plate by using a wooden stick (samples that could not be picked up with a stick were transferred with a 1-µL pipette). It was covered with 1 µL of 70% formic acid and allowed to dry at room temperature.

### Differential centrifugation with PE

Five milliliters of blood culture fluid from a positive blood culture bottle was transferred to a plain (red top, no additive) blood tube. The tube was then centrifuged at room temperature for 10 seconds at 1,610 g (3,000 rpm) to remove cellular elements (leukocytes and erythrocytes). The supernatant was carefully transferred to a new plain tube using a Pasteur pipette. One milliliter of distilled water was added, and the mixture was vortexed. To obtain the bacterial pellet, the tube was centrifuged for 10 minutes at 3,007 g (4,100 rpm). The supernatant was then gently decanted and removed. To prepare for ethanol/formic acid protein extraction, 300 µL of distilled water was added to the pellet, and the contents were vortexed. This mixture was then transferred to a 1.5-mL microcentrifuge tube by using a pipette. (In this step, additional washing was performed before adding ethanol to the samples that were still rich in erythrocytes). Subsequently, 900 µL of 96% ethanol was added to the tube and vortexed, followed by centrifugation at 18,188 g (14,000 rpm) for 3 minutes at room temperature. The supernatant was removed by using a pipette, and the remaining alcohol was allowed to evaporate at room temperature for 5 minutes. Depending on the pellet quantity (15 µL for a small-sized pellet, 20 µL for a medium-sized pellet, and 25 µL for a large-sized pellet), an appropriate amount was chosen, and both 70% formic acid and acetonitrile (ACN) were added in the same quantity (15–25 µL). The mixture was homogenized with a pipette tip until fully mixed. After thorough vortexing, the tube was centrifugated at 18,188 g for 3 minutes. One microliter of the resulting supernatant was transferred onto the target plate in two spots for subsequent drying (the result with the higher score was accepted).

### Analysis with MALDI-TOF MS

At the end of all three preparation methods, after the drying process was completed on the target plate, 1 µL of the α-cyano-4-hydroxycinnamic acid (HCCA) matrix solution (Bruker Daltonics GmbH, Bremen, Germany) was applied to the spots on the target plate. After drying, it was analyzed with the Bruker Biotyper (Microflex LT/SH Smart) MALDI-TOF MS using the MBT Compass analysis software standard and the Sepsityper module (MALDI BioTyper Library ver. 11.0.0.0). The spectral data were matched to the database for identification. For points where the result could not be obtained, at least 200 laser shots were collected manually. In each study, the *Escherichia coli* ATCC 25922 strain was used as a control. For the standard module and Sepsityper module, correct genus level identification scores were ≥ 1.70 and ≥ 1.60, respectively. Scores ≥ 2.0 and ≥ 1.80 were regarded as reliable identification at species level.

### Statistical analysis

In the study, the sample size calculation for the McNemar chi-square test was conducted with a statistical power of 95%, with a Type 1 error rate of 5%, a moderate effect size of 0.3, and 3 degrees of freedom, resulting in a required sample size of 191 (Source: GPower). The study included more samples (*n* = 240) than the required sample size. McNemar analysis was used to compare the performance of the MBT Sepsityper IVD Kit, SDS lysis, and differential centrifugation with PE methods against that of routine colony identification. The analysis also compared the performance of the methods using the standard and Sepsityper modules. Statistical significance was determined with *P* value < 0.05. The data were analyzed using the Microsoft Office Excel 2016 and IBM SPSS Statistics 27.

## RESULTS

### Sample distribution

The study included 245 blood culture samples obtained from positive blood culture bottles. Among these, five samples showed polymicrobial growth and were excluded from the study. Of the total 240 samples included in the study, 124 (51.7%) were identified as Gram-negative and 116 (48.3%) as Gram-positive growth according to the reference method.

### Performance evaluation

**Fig 2:**
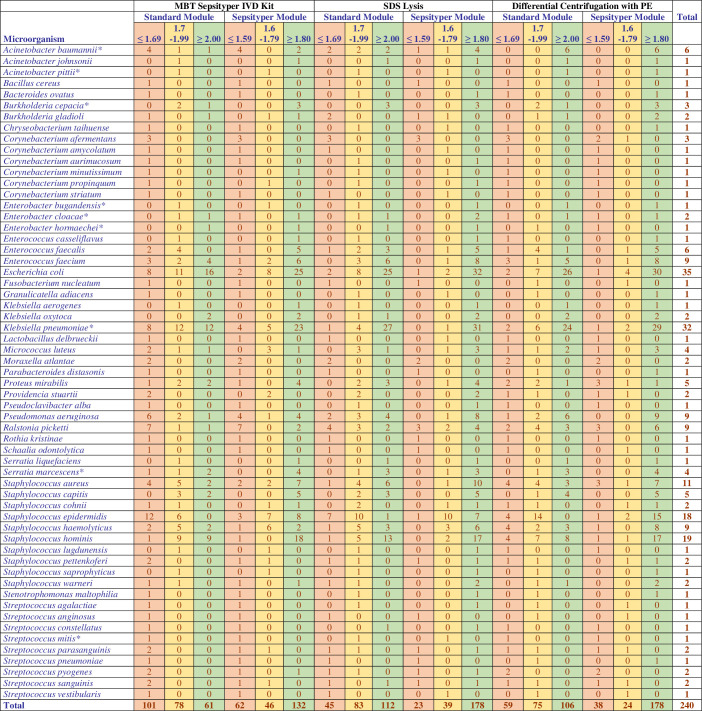
Correct identification performance according to the score ranges obtained in two different analysis modules of the three methods. By analysing the Sepsityper module, four *Staphylococcus* isolates were identified correctly at genus level and incorrectly at species level. Since the 1.6–1.79 score range in the Sepsityper module represented correct identification at genus level, they were included in the correct identification in this score range in the table. Eight isolates were misidentified in the Sepsityper module; they were included in the “no identification” group, which represents a score range of ≤ 1.59 in the table. *As members of the *Acinetobacter baumannii* complex, *Enterobacter cloacae* complex, *Burkholderia cepacia* complex, *Serratia marcescens/S. nematodiphila/S. ureilytica*, *Klebsiella pneumoniae/K. variicola*, and *Streptococcus mitis* group could not be clearly differentiated by MALDI-TOF MS (16), the result was considered correct when any of these members were identified.

In the study, the direct bacterial identification performance of 240 blood culture samples with monomicrobial growth was determined by applying the MBT Sepsityper IVD Kit, SDS lysis, and differential centrifugation with PE protocols, and the results were compared with the identification results obtained by the routine colony identification method using MALDI-TOF MS. A total of 59 species were considered for evaluation. The correct identification performance of the three methods, based on score intervals and analyzed with the standard and Sepsityper module, is presented in Fig. 2.

### Comparison of the three methods by using the standard module

At the species level (score ≥2), the highest rate of accurate identification was observed with the SDS lysis method in 112 samples (46.7%), followed by the differential centrifugation with PE method in 106 samples (44.2%), and the Sepsityper kit in 61 samples (25.4%).

At the genus level (score range 1.7–1.99), the highest accuracy was achieved with the SDS lysis method in 83 samples (34.6%), followed by the Sepsityper kit in 78 samples (32.5%), and the differential centrifugation with PE method in 75 samples (31.3%).

In total, 101 samples (42.1%) could not be identified at the species or genus levels using the Sepsityper kit, 59 samples (24.6%) by the differential centrifugation with PE method, and 45 samples (18.8%) by the SDS lysis method (score ≤1.69).

By using the standard module, the SDS lysis method (*n* = 195) achieved a higher number of correct identifications at the species and genus levels together compared to the differential centrifugation with PE method (*n* = 181) and the Sepsityper kit (*n* = 139). There was no statistically significant difference between the SDS lysis method and the differential centrifugation with PE method (*P* = 0.098). However, a significant difference was observed between the SDS lysis method and the Sepsityper kit, as well as between the differential centrifugation with PE method and the Sepsityper kit (*P* < 0.001, both).

### Comparison of the three methods by using the Sepsityper module

At the species level (score ≥1.8), the highest rate of accurate identification was observed with both the differential centrifugation with PE method and SDS lysis method, each with 178 samples (74.2%). The Sepsityper kit achieved correct identification at species level in 132 samples (55%).

At the genus level (score range 1.6–1.79), the highest accuracy was observed with the Sepsityper kit in 46 samples (19.2%), followed by the SDS lysis method with 39 samples (16.3%), and the differential centrifugation with PE method with 24 samples (10%).

In total, 62 samples (25.8%) could not be identified at the species or genus levels by the Sepsityper kit, 38 samples (15.8%) using the differential centrifugation with PE method, and 23 samples (9.6%) by the SDS lysis method (score ≤1.59).

By using the Sepsityper module, the SDS lysis method (*n* = 217) resulted in a higher number of correct identifications at the species and genus levels together compared to the differential centrifugation with PE method (*n* = 202) and the Sepsityper kit (*n* = 178). This difference was found to be statistically significant (*P* = 0.028 and *P* < 0.001). A significant difference was also observed between the differential centrifugation with PE method and the Sepsityper kit (*P* < 0.001).

The performance of the methods for accurate identification is summarized in Table 1.

**TABLE 1:** Performance of the methods for accurate identification according to species and genus levels

	Sepsityper kit		SDS lysis		Centrifugation + PE	
Total (*n* = 240)	Standard module	Sepsityper module	Standard module	Sepsityper module	Standard module	Sepsityper module
Species level (high confidence)	61 (25.4%)	132 (55%)	112 (46.7%)	178 (74.2%)	106 (44.2%)	178 (74.2%)
Genus level (low confidence)	78 (32.5%)	46 (19.2%)	83 (34.6%)	39 (16.3%)	75 (31.3%)	24 (10%)
Correct identification^*a*^ (high and low)	139 (57.9%)	178 (74.2%)	195 (81.3%)	217 (90.4%)	181 (75.4%)	202 (84.2%)
No identification	101 (42.1%)	62 (25.8%)	45 (18.8%)	23 (9.6%)	59 (24.6%)	38 (15.8%)

^a^ Numbers of correct identification according to species and genus levels together.

### Comparison of the standard module with the Sepsityper module

Of the 240 samples analyzed with all three preparation methods, the Sepsityper module had significantly higher numbers of overall correct identification at species level results than the standard module (*P* < 0.001).

Correct identification of Gram-negative bacteria increased with the Sepsityper module compared with the standard module from 63.7% to 75.8% by the Sepsityper kit, from 86.3% to 91.9% by SDS lysis and from 87.9% to 89.5% by differential centrifugation with PE (Table 2). The difference between the modules in the correct identification of Gram-negative bacteria at the species level was found to be statistically significant in both SDS lysis and Sepsityper kit (*P* = 0.039 and *P* < 0.001, respectively). There was no statistically significant difference between the modules in the differential centrifugation with PE method (*P* = 0.727).

**TABLE 2:** Numbers and percentages of correctly identified samples at species level in Gram-positive and Gram-negative bacteria by three methods and two different analysis modes

	Sepsityper kit		SDS lysis		Centrifugation + PE	
	Standard module	Sepsityper module	Standard module	Sepsityper module	Standard module	Sepsityper module
Gram-negative bacteria (*n* = 124)	79 (63.7%)	94 (75.8%)	107 (86.3%)	114 (91.9%)	109 (87.9%)	111 (89.5%)
Gram-positive bacteria (*n* = 116)	60 (51.7%)	82 (70.7%)	88 (75.9%)	101 (87.1%)	72 (62.1%)	91 (78.4%)
Total (*n* = 240)	139 (57.9%)	176 (73.3%)	195 (81.3%)	215 (89.6%)	181 (75.4%)	202 (84.2%)

These ratios for Gram-positive bacteria were from 51.7% to 70.7% by the Sepsityper kit, from 75.9% to 87.1% by SDS lysis, and from 62.1% to 78.4% by differential centrifugation with PE. The difference between the modules in the correct identification of Gram-positive bacteria at the species level was found to be statistically significant in all three methods compared (*P* < 0.001).

### Distribution by score ranges

Analysis rates by score ranges are provided in Table 3. Among the samples included in the study, the highest percentage of analysis with a score of ≥2 was observed by the differential centrifugation with PE method in the Sepsityper module at 56.7% (136/240). The highest rate of a score of ≤1.59 was primarily associated with the Sepsityper kit analyzed by the standard module, occurring at a rate of 34.2% (82/240).

**TABLE 3:** Numbers and percentages of correctly identified samples at species level based on MALDI-TOF MS score ranges^*a*^

	Sepsityper kit		SDS lysis		Centrifugation + PE	
	Standard module	Sepsityper module	Standard module	Sepsityper module	Standard module	Sepsityper module
≥ 2	61 (25.4%)	69 (28.8%)	112 (46.7%)	122 (50.8%)	105 (43.8%)	136 (56.7%)
1.8–1.99	55 (22.9%)	63 (26.3%)	60 (25%)	56 (23.3%)	51 (21.3%)	42 (17.5%)
≥ 1.8	116 (48.3%)	132 (55%)	172 (71.7%)	178 (74.2%)	156 (65%)	178 (74.2%)
1.7–1.79	23 (9.6%)	25 (10.4%)	23 (9.6%)	22 (9.2%)	25 (10.4%)	15 (6.3%)
1.6–1.69	19 (7.9%)	21 (8.8%)	8 (3.3%)	15 (6.3%)	8 (3.3%)	9 (3.8%)
1.6–1.8	42 (17.5%)	46 (19.2%)	31 (12.9%)	37 (15.4%)	33 (13.8%)	24 (10%)
≤ 1.59 (unidentified)	82 (34.2%)	62 (25.8%)	37 (15.4%)	25 (10.4%)	51 (21.3%)	38 (15.8%)
Total	240 (100%)	240 (100%)	240 (100%)	240 (100%)	240 (100%)	240 (100%)

^*a*^ Misidentifications at species and genus levels were included in the “≤ 1.59 (unidentified)” group.

The Sepsityper module uses a lower cutoff score for identification than the standard module. When using a score of 1.8 instead of the default score of 2.0, the results obtained from the standard module showed correct identification rates at the species level of 48.3% with the Sepsityper kit, 71.7% with SDS lysis, and 65% with differential centrifugation with PE. When a score of 1.6 was used instead of the default score of 1.7, the unidentified rates were 34.2%, 15.4%, and 21.3%, respectively. The correct identification rate with the standard module was clearly below that of the Sepsityper module, despite these two adjusted scores.

### Misidentification

Eight genus-level and four species-level misidentifications were detected in studies performed by the Sepsityper kit and SDS lysis method only by analyzing the Sepsityper module in the range of 1.60–1.79 scores. Misidentifications at the genus level were observed in both Gram-negative and Gram-positive bacteria (Table 4). The species-level misidentifications were all in *Staphylococcus* isolates.

**TABLE 4:** Distribution of misidentified microorganisms

Microorganism	Misidentification at genus level	Method	Score
*Staphylococcus epidermidis*	*Lactobacillus helveticus*	Sepsityper Kit	1.61
*Ralstonia picketti*	*Sinomonas atrocyanea*	Sepsityper Kit	1.61
*Corynebacterium afermentans*	*Gluconobacter oxydans*	Sepsityper Kit	1.64
*Acinetobacter baumannii*	*Companilactobacillus alimentarius*	Sepsityper Kit	1.60
*Pseudoclavibacter alba*	*Paucilactobacillus oligofermentans*	Sepsityper Kit	1.62
*Ralstonia picketti*	*Klebsiella pneumoniae*	Sepsityper Kit	1.70
*Streptococcus constellatus*	*Ligilactobacillus salivarius*	Sepsityper Kit	1.63
*Corynebacterium afermentans*	*Trichomonascus ciferrii*	SDS	1.60

Microorganism	Misidentification at species level	Method	Score
*Staphylococcus haemolyticus*	*Staphylococcus simiae*	SDS	1.63
*Staphylococcus epidermidis*	*Staphylococcus aureus*	Sepsityper Kit	1.79
*Staphylococcus epidermidis*	*Staphylococcus aureus*	SDS	1.64
*Staphylococcus epidermidis*	*Staphylococcus aureus*	Sepsityper Kit	1.60

### The organisms that could not be identified

The majority of the unidentified microorganisms were Gram-positive bacteria. Neither the Sepsityper kit nor the differential centrifugation with PE method by the standard module could identify any *Corynebacterium* species. Similarly, none of the *Streptococcus* species were identified by the standard module with the Sepsityper kit. Overall, a decrease in the number of unidentified microorganisms was observed with the Sepsityper module. The distribution of unidentified microorganisms is presented in Table 5 according to the three methods and analysis modules.

**TABLE 5:** Distribution of unidentified microorganisms according to the three methods and analysis modules

Microorganism	Isolate count (*n* = 240)	Sepsityper kit		SDS lysis		Centrifugation + PE	
		Standard module	Sepsityper module	Standard module	Sepsityper module	Standard module	Sepsityper module
		≤ 1.69	≤ 1.59	≤ 1.69	≤ 1.59	≤ 1.69	≤ 1.59
*Enterobacterales*	86	20	8	3	1	8	8
*Pseudomonas aeruginosa*	9	6	4	2	0	1	0
*Acinetobacter* spp.	8	4	4	2	1	0	0
Other Gram-negatives	21	15	14	10	8	6	5
Gram-negative total	124	45	30	17	10	15	13
*Staphylococcus aureus*	11	4	2	1	0	4	3
CNS^*a*^	59	19	7	11	2	15	4
*Streptococcus* spp.	12	12	9	6	5	6	6
*Enterococcus* spp.	16	5	2	1	0	5	0
*Corynebacterium* spp.	8	8	6	5	3	8	7
Other Gram positives	10	8	6	4	3	6	5
Gram-positive total	116	56	32	28	13	44	25
Unidentified total		101	62	45	23	59	38

^*a*^ Coagulase-negative staphylococci.

### Turnaround time

Each of the three preparation methods studied required approximately 30–40 minutes for the direct identification of bacterial isolates from a single blood culture bottle by MALDI-TOF MS.

## DISCUSSION

The current gold standard for diagnosing bloodstream infection is through conventional blood culture, which takes at least 48 hours. However, septicemia has a high mortality rate, and every hour between a patient’s registration and antibiotic administration is associated with a 9% increase in mortality (17). Although molecular methods and commercial kits are available for identification, they are not widely accessible due to cost and practical limitations (18). For all that, clinical benefit could be achieved in 24% of cases with early identification directly from blood culture by MALDI-TOF MS (19).

A study found that the minimum concentration of microorganisms needed for identification using MALDI-TOF MS was >10^7^–10^8^ in simulated blood culture bottles and >10^6^ on the target plate (20). There was no significant difference between Gram-positive and Gram-negative bacteria. However, identifying microorganisms directly from blood culture samples is challenging due to the separation of microorganisms from blood cell components and culture fluid content. While there are commercial kits available, most protocols are “in-house” and involve various extraction steps and detergents (6, 13, 21, 22). Studies using centrifugation alone or a serum separator tube had sensitivities ranging from 43% to 95% (10, 12, 23–26). Sensitivities ranged from 53% to 86% when using different detergents, such as saponin, SDS, ammonium chloride, or Triton X-100 (9, 19, 22, 24, 27). Additional extraction steps using ethanol, formic acid, or acetonitrile further increased sensitivities from 49% to 88.9% (14, 28–30). The time to obtain results varied depending on the extraction and centrifugation steps, ranging from 10 to 45 minutes (6, 7, 26, 29). This process took longer for Gram-positive isolates compared to the rapid identification method (31).

In a 2009 paper, Scola and Raoult (32) predicted that direct identification would replace conventional bacterial identification in blood cultures but highlighted two challenges. First, only one agent may be identified or misidentified in cases of polymicrobial growth, so Gram staining should be performed in all cases. Second, there is inadequate identification of viridans streptococci. Despite these challenges, Scola and Raoult’s research has spurred the development of new methods and software for identifying positive blood culture bottles using MALDI-TOF MS in the field of clinical microbiology. The problem of identification in Gram-positive bacteria and polymicrobial growths persists in later studies, including the present one.

A review study by Morgenthaler and Kostrzewa (8) evaluated the Sepsityper kit. The study analyzed 21 previous studies and reported an overall correct identification rate of 80% at the species level, with higher rates for Gram-negative bacteria (90%) compared to Gram-positive bacteria (76%) from 3,320 positive blood culture bottles. However, it has been noted that some researchers used lower identification scores. In our study, the identification rate in the Sepsityper module with the Sepsityper kit was 55% at the species level and 74.2% at the genus level, and 70.7% of Gram-positive and 75.8% of Gram-negative bacteria were correctly identified at the species level. Differing results between studies may be due to two different workflows of the Sepsityper kit, researchers using different scores, multiple transfers from the pellet to the wells on the target plate, and the revision of MALDI-TOF MS libraries over the years.

Kayin *et al.* (6), compared the standard and Sepsityper module analyses with three preparation methods and drew attention to the failure in identification rates for streptococci and enterococci in all three methods they analyzed, as well as for staphylococci and micrococci in the centrifugation method. In our study, unidentified microorganisms were mostly seen in the Sepsityper kit, and it is noteworthy that none of the *Streptococcus* and *Corynebacterium* species could be identified in the analysis performed with the standard module of the Sepsityper kit. Even in *Enterobacterales* isolates, which were expected to be correctly identified the most, 23% could not be identified in the standard analysis of the Sepsityper kit, while this rate decreased to 9% with the Sepsityper module. Additionally, even when the results obtained from the standard module were re-evaluated using scores of 1.8 and 1.6, as in the Sepsityper module, instead of the default scores of 2.0 and 1.7, the correct identification rate obtained with the standard module was still lower than that with the Sepsityper module in all three preparation methods. In addition to providing a lower identification score, the Sepsityper module also allows the sample to be registered as “Sepsityper sample type”. Thus, a certain data processing of the module, taking into account undesired mass spectrum peaks originating from blood cells (6), may have resulted in this rate increase.

In our study, eight genus-level and four species-level misidentifications were detected with the Sepsityper kit and SDS lysis method only by analyzing the Sepsityper module. In the study of Perše *et al*. (21) , the misidentification of the samples was attributed to inadequate cleaning and the presence of residual material on the reusable target plate. The fact that the target plate is cleaned with ethanol and trifluoroacetic acid in our laboratory and the misidentification is only seen with the Sepsityper software module and not with the standard module, which distracts us from the problem of plate cleaning. Misidentifications of our study may indicate that the Sepsityper software needs to be improved, either because the Sepsityper software gives reliable correct identification results with lower scores or because the library matching is not compatible with the resulting spectra.

We included SDS in our study because it is ionic and a potent agent in cell lysis. In the literature, there are very few studies on MALDI-TOF MS directly from positive blood culture with the SDS lysis method, and although it is not a standardized method, correct identification rates (score ≥1.7) vary between 72% and 96% (9, 12, 19, 22, 30, 33). In a study comparing the two detergents, the rate of correct identification with SDS was significantly higher than with saponin (9).

Since a detergent was not used in the differential centrifugation method, ethanol/formic acid extraction was performed in addition to centrifugation in our study to increase the identification rates. The negatives of the method include more steps and longer working time. In two different studies using the differential centrifugation with ethanol/formic acid protein extraction method from positive blood cultures, correct identification rates (score ≥ 1.7) were found to be 87% in one (10) and 65% in the other (12). The rate we found in our study is between the rates detected in these two studies.

A systematic review and meta-analysis conducted by Ruiz-Aragón *et al*. (34) in 2018 aimed to assess the accuracy of different methods for directly identifying bacteria from positive blood culture bottles using MALDI-TOF MS. The analysis included 32 studies conducted between 2011 and 2015. Overall, the quality of the studies was moderate. The results showed that the accurate identification rates for Gram-positive bacteria ranged from 17% to 98%, with a cumulative rate of 72%. For Gram-negative bacteria, the correct identification rates varied from 66% to 100%, with a cumulative effect of 92%. The study concluded that MALDI-TOF MS had high accuracy in identifying Gram-negative bacteria, particularly *Enterobacteriaceae*, at a rate of 96%, while its accuracy for identifying Gram-positive bacteria directly from blood culture bottles was moderate, with some species having lower accuracy. The analysis revealed that the rates of correct identification varied widely depending on the methods and centers used.

In the study by Zhou *et al*. (22), gene sequencing was performed on all viridans group streptococci (20 samples in total) and microorganisms, with inconsistent results between direct and colony MS identification. Of these samples, 15 (75%) had molecular sequencing results consistent with colony MS results, one was consistent with direct MS results (*Streptococcus mitis*), and the remaining four (one *Streptococcus sinensis*, one *Streptococcus mitis,* and two *Streptococcus tigurinus*) were inconsistent with both colony and direct MS results. This supports the problem of MALDI-TOF MS in the correct identification of microorganisms in the *Streptococcus mitis* group. In our study, isolates with discordant identification results could not be sequenced, and this is one of the limitations of our study. In the study by Kok *et al*. (35) Gram-negative bacteria were correctly identified significantly more than Gram-positive bacteria at the species level. This was the same situation in our study as in other studies. This may be attributed to the thick cell wall structure that makes protein extraction more difficult, the difficulty of removal from serum due to the adhesion of bacteria to erythrocytes, and the formation of smaller pellet volumes due to the slower growth of Gram-positive bacteria in positive blood cultures (6, 21).

Other limitations of our study include the evaluation of a single blood culture system, the participation of only one center, and the exclusion of yeasts. Despite these limitations, we think that our study provides important data since it includes 240 blood culture samples and a large number of bacterial species.

In conclusion, the best performance among the three methods compared was achieved with the SDS lysis method. Of the two analysis modules compared, better performance was achieved with the Sepsityper software module, although the risk of misidentification by this module should not be ignored. In the diagnosis of sepsis, conventional identification remains the gold standard as it allows for the detection of multiple microorganisms and performing antibiograms. However, laboratories can contribute to empirical treatment by identifying sepsis agents early if they apply the direct identification method by MALDI-TOF MS from positive blood cultures in addition to culture, within the framework of their own possibilities.
